# Supplementary material for: Neural signals regulating motor synchronization in the primate deep cerebellar nuclei
Source: Nat Commun. 2022 May 6;13:2504. doi: 10.1038/s41467-022-30246-2 (PMC9076601; doi:10.1038/s41467-022-30246-2)
Supplement: Supplementary file 1 — Supplementary Information [file 41467_2022_30246_MOESM1_ESM.pdf]

Supplementary information for

Neural signals regulating motor synchronization in the primate deep cerebellar nuclei

by Ken-ichi Okada, Ryuji Takeya, Masaki Tanaka

Supplementary Methods

Supplementary Movies 1–2

Supplementary Figures 1–15

Supplementary Methods

Data analysis for Supplementary Figures and relevant text

Mutual information (related to Supplementary Fig. 3d and e).

In addition to the directional index (DI) and the prediction index (PI), the ability of each neuron to discriminate saccade direction (ipsilateral or contralateral) or task condition (synchronized or reactive) was also evaluated by calculating the mutual information (MI)[1,2]. For this analysis, neuronal activity was measured from the spike density function during 100 ms before (Unilateral and Bilateral neurons) or after (Postsaccade neurons) saccades. The MI was defined as,

$$MI = \sum_{R}\sum_{C} P(c,r)log_2\left(\frac{P(c,r)}{P(c)P(r)}\right)$$

where *P(c, r)* was the joint probability of the task condition *c* and the neuronal response *r* (higher or lower than the median). For the comparison with the DI and PI, the sign of the MI was assigned so that the preference for ipsilateral saccades or synchronized task was positive and the preference for contralateral saccades or reactive task was negative.

Temporal scaling (related to Supplementary Fig. 4).

As shown in Fig. 2b and c, both Unilateral and Bilateral neurons exhibited ramping activity before saccade execution, which might be temporally scaled for different SOAs. To quantify temporal scaling capability of each group of neurons, we compared (1) the timing of the peak activity, (2) the magnitude of the peak activity, and (3) the slope of the ramping profile measured from the spike density function aligned with saccades across the three SOA conditions. Each parameter was tested by repeated measures of ANOVA ($P < 0.05$). We also examined the slope of the time-scaled data normalized for the SOA (Supplementary Fig. 4c). The same analysis was also applied to the spike density functions (σ = 30 ms) obtained from individual trials

(Supplementary Fig. 4d)[3].

Decoding analysis (related to Supplementary Fig. 9)

To evaluate the neuronal coding for saccade timing, population decoder was constructed for each group of neurons using the Neural Decoding toolbox for Matlab[4]. For comparison, we used the same set of the data as the partial correlation analysis in Figs. 3 and 4 (synchronized saccades in the preferred direction, 550 ms SOA). The decoder identified whether the ISI was shorter or longer than the median (timing of the next saccade, Supplementary Fig. 9a) or whether the saccade preceded or lagged the target onset (temporal error, Supplementary Fig. 9b). The classification accuracy was computed for every 100-ms interval (10 ms step) after saccades. Since we recorded only single neuron at a given moment, pseudo-population data for each neuron group were created by randomly resampling trials in the same condition. For each decoding run, the data was divided into five sets of trials, and the maximum correlation coefficient classifier was trained on the four sets and tested for the remaining. The mean population vector of normalized neuronal activity in the training set was used as a template for classification. The classifier calculated Pearson's correlation coefficient between the test data and the template, and the condition with the highest correlation was taken as the predicted result. This procedure was repeated five times for cross-validation by swapping the training and test sets. Classification accuracy was defined as the average percentage of correct trials identified in the test set during cross-validation. These procedures were repeated 100 times with different pseudo-population data, and the mean values are reported in Supplementary Fig. 9. To evaluate whether the decoder performance was better than chance, the permutation test (100 repeats) was performed by shuffling the relationship between neuronal activity and saccade timing. The result of the permutation test for each time point (10 ms) is shown by the solid bar below the traces ($P < 0.05$). We also constructed the decoder for tempo (Supplementary Fig. 9c, based on the data of synchronized saccades in the preferred direction with different SOAs), task condition

(Supplementary Fig. 9d, synchronized and reactive saccades in the preferred direction with different SOAs), and saccade direction (Supplementary Fig. 9e, synchronized saccades in both directions with different SOAs) using the same classification method (maximum correlation coefficient classifier).

Comparison of neuronal activity related to sensory versus motor events.

Correlation analysis (related to Supplementary Fig. 10c)

The neuron shown in Fig. 5b exhibited a strong transient activity that peaked just before synchronized saccades (> 5th saccades, bottom trace with orange shading), but the activity bottomed out around the time of the second saccade in the sequence (red trace with orange shading), indicating that the neuronal activity did not always coincide with saccades. In addition to the sensorimotor index (SMI) that compared the magnitude of neuronal activity for sensory versus motor alignments, we also quantified the similarity of the time courses of neuronal activity between the second and the later saccades by calculating the correlation of the spike density profiles (± 275 ms aligned either with the target onset or saccades). The correlation of the saccade-aligned data for Bilateral neurons was significantly smaller than that for Unilateral neurons, while the correlation of the target-aligned data showed an opposite trend. These results further support that the activity of Bilateral neurons was related to sensory prediction rather than saccade execution.

Time warping analysis (related to Supplementary Fig. 11a)

To further clarify whether the neuronal activity was better aligned to sensory prediction or motor execution, we performed the time warping analysis modified from Perez et al. (2013)[5]. We used only the data during the transition from reactive to synchronized saccades (a 700-ms period starting from 200 ms before the second target onset). To quantify the trial-by-trial variability in neuronal activity associated with target onset (Ls), we calculated the likelihood of

spike occurrence on each trial using the normalized spike density function obtained from the remaining trials as a probability distribution. Next, the data were transformed to be aligned with saccades. Saccade timing in each trial was time-warped to the mean of all trials, and spike trains before and after the saccade was accordingly stretched or shrank in time without changing the total length of the data (700 ms). Then, we computed the likelihood of spike occurrence associated with motor execution (Lm). The relative magnitude of the trial-by-trial variability for the sensory and motor alignments was evaluated by calculating the time-warping index (TWI) defined as,

$$TWI = \log_{10}(Ls/Lm)$$

If the neuronal activity was better aligned with the target onset, the TWI for early reactive saccades would have a positive value. For comparison, we also calculated the TWI for synchronized saccades (a 700-ms period starting from 350 ms before the sixth and subsequent target onset). These data are shown in Supplementary 11a in comparison with the sensorimotor index (SMI) calculated for the second saccades.

ROC analysis (related to Supplementary Fig. 11b)

The activity of saccade-related neurons initially lagged behind the target onset but later preceded it as the trial progressed (Fig. 5a). To examine the changes in the time course of neuronal activity for the second versus later targets (> 5th), we quantified the difference in phase of the spike density functions aligned with the target onset using the receiver operating characteristics (ROC) analysis. The ROC curve was constructed by comparing the normalized spike density profiles during the first and the later cycles for different levels of threshold that spanned the entire time range (1,100 ms). The area under the ROC curve (AUC) was greater than 0.5 if the neuronal activity in the first cycle lagged behind that in later cycles, and less than 0.5 otherwise.

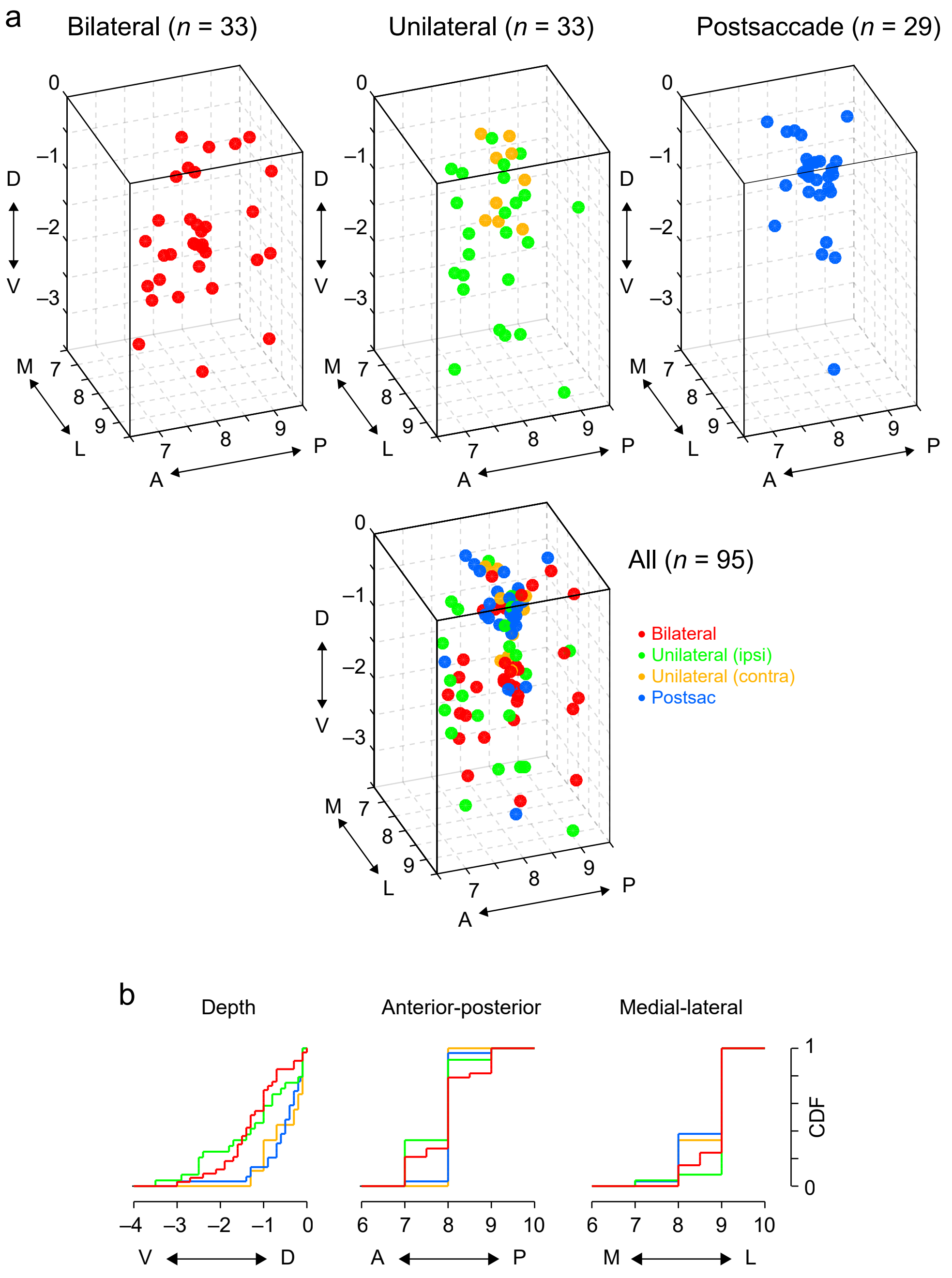

Supplementary Figure 1 | Location of all recorded neurons. (**a**) Three-dimensional reconstruction of recording sites for each type of neurons (top panels) and all neurons together (bottom). Different colours indicate different types of neurons. Anteroposterior and mediolateral locations are based on the stereotaxic coordinates of electrode penetrations. Dorsoventral locations are relative to the physiologically-identified dorsal border of the cerebellar nucleus. (**b**) Normalized cumulative distributions of the different types of neurons along the cardinal axes. Note that Unilateral neurons with an ipsilateral preferred direction (green) and Bilateral neurons (red) widely distributed along the dorsoventral axis (depth), whereas Unilateral neurons with a contralateral preferred direction (orange) and Postsaccade neurons (blue) tended to be located dorsally.

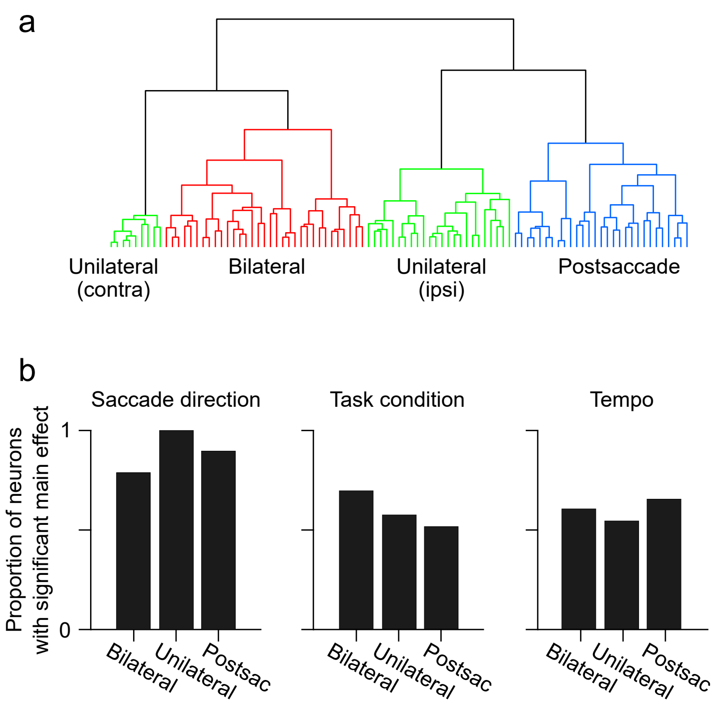

Supplementary Figure 2 | Classification of neurons and their basic response properties. (**a**) A dendrogram derived from the cluster analysis. Neurons were classified into four groups based on the time course of neuronal activity aligned with synchronized saccades (± 275 ms for both directions, Fig. 2a). In the subsequent analyses, the data of Unilateral neurons with different directional preferences were combined. Red, green, and blue clusters indicate Bilateral ($n = 33$, $n = 22$ and 11 for monkeys I and J, respectively), Unilateral ($n = 33$, $n = 28$ and 5 for I and J, respectively), and Postsaccade neurons ($n = 29$, $n = 22$ and 7 for I and J, respectively), respectively. (**b**) Results of 3-way ANOVA for each type of neurons. Neuronal activity during 200 ms before (Bilateral and Unilateral neurons) or after (Postsaccade neurons) saccades for each neuron was compared across three factors including saccade direction, task condition (synchronized vs. reactive), and the SOA. Each black bar indicates the proportion of neurons with a significant main effect ($P < 0.05$). Consistent with our cluster grouping, all Unilateral neurons showed main effect of saccade direction and many Bilateral neurons showed main effect of task condition. Note that ANOVA only considers significant differences in neuronal activity at specific time intervals and does not consider the time course or relative magnitude of neuronal activity between conditions.

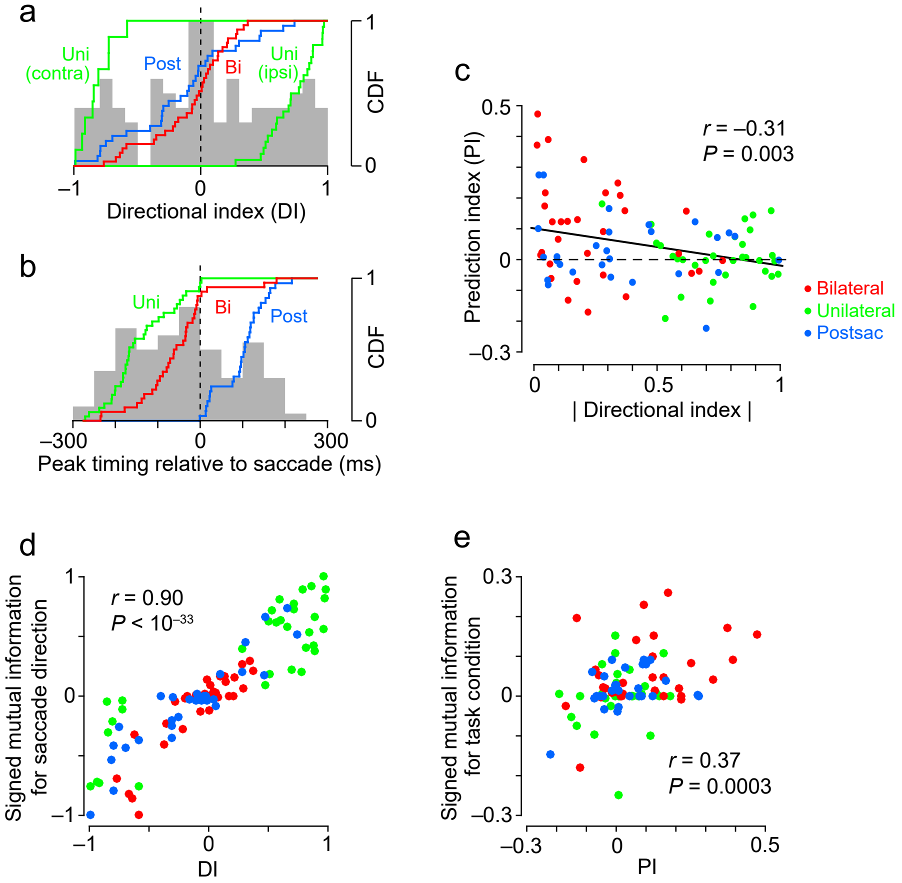

Supplementary Figure 3 | Modulation of neuronal activity for different saccade direction and task condition. (**a**) Distribution of the directional index (DI) for each neuron type. (**b**) Timing of peak activity in the different types of neurons. The values between any pair of neuron types are statistically different (Tukey test, $P < 0.01$). Timing of peak activity was measured from the spike density profile aligned with synchronized saccades in the preferred direction. (**c**) Relationship between the DI and PI calculated for the data of 550-ms SOA. There was a significant negative correlation between the DI and PI for all neurons ($r = -0.31$, $P = 0.003$), indicating that neurons showing weak directionality tended to exert greater activity for synchronize than reactive saccades. A two-way ANOVA (neuron types × monkeys) for each index showed no significant difference between monkeys (DI, $F_{1,94} = 0.04$, $P = 0.84$; PI, $F_{1,90} = 0.1$, $P = 0.76$; peak timing, $F_{1,93} = 0.8$, $P = 0.37$). (**d**) Comparison of the mutual information for saccade direction (Supplementary Methods) with the DI. The mutual information is signed so that the preference for ipsilateral saccades is negative. These two indices significantly correlated ($r = 0.90$, $P = 1.8 \times 10^{-34}$). As with the DI, the mutual information did not differ between Bilateral and Postsaccade neurons (Tukey test, $P = 0.56$), while mutual information for Unilateral neurons differed from that for the others ($P < 0.01$). (**e**) Comparison between the mutual information for task condition and the PI. The mutual information is signed so that the preference for the reactive saccade task is negative. These two indices were significantly correlated ($r = 0.37$, $P = 0.0003$). As in the PI, only the mutual information for Bilateral neurons was significantly different from zero (one sample t-test, two-sided, $t_{30} = 3.36$, $P = 0.002$).

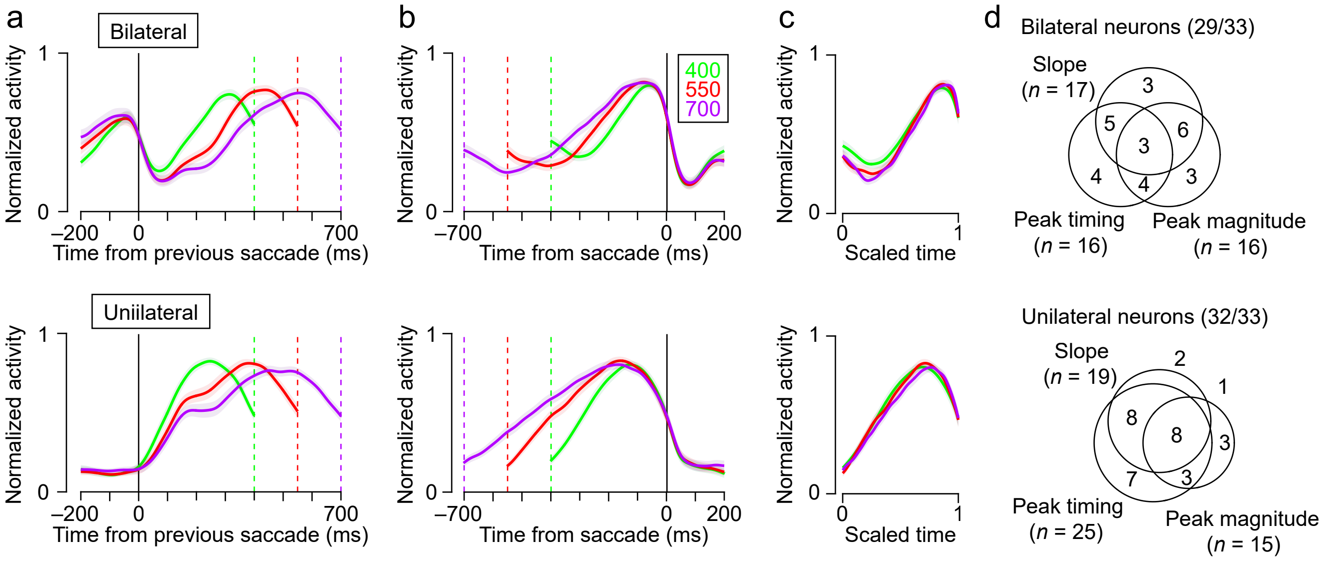

Supplementary Figure 4 | Temporal scaling capability of ramping activity in Bilateral and Unilateral neurons. (**a**) Normalized population activity (mean ± s.e.) of Bilateral and Unilateral neurons during synchronized saccades with different SOAs. The data are aligned with saccades in the non-preferred direction (solid black vertical line) and terminate after 400, 550 or 700 ms for each relevant SOA condition (dashed vertical lines). (**b**) Normalized activity (± s.e.) aligned with synchronized saccades in the preferred direction. In the population, both Bilateral and Unilateral neurons showed significant changes in the slope of ramping activity depending on the SOAs (one-way ANOVA, $F_{2,32} = 36.9$, $P = 3.1 \times 10^{-11}$ for Bilateral neurons, $F_{2,32} = 23.9$, $P = 2.5 \times 10^{-8}$ for Unilateral neurons) but not in the magnitude of the peak activity ($F_{2,32}$ =1.64, $P = 0.20$ for Bilateral neurons, $F_{2,32} = 0.46$, P = 0.63 for Unilateral neurons). Unilateral neurons only showed a significant change in the timing of the peak activity ($F_{2,32} = 18.7$, $P = 4.1 \times 10^{-7}$). (**c**) Temporally-scaled normalized activity for the data in **b**. The slopes of ramping activity in the scaled data did not differ across the SOAs for both Bilateral and Unilateral neurons (one-way ANOVA, Bilateral, $F_{2,32}$ = 0.39, $P = 0.68$; Unilateral, $F_{2,32} = 0.17$, $P = 0.84$). The error band indicates ± s.e. (**d**) Counts of neurons with significant changes either in the slope of ramping activity, the timing or magnitude of the peak activity. Most Bilateral (29/33) and Unilateral (32/33) neurons changed either of the three parameters (ANOVA, $P < 0.05$). For almost all neurons, these changes were scaled orderly except for a few neurons exhibiting a selective change only for the 550-ms SOA (one Unilateral neuron for peak timing, one Unilateral and two Bilateral neurons for peak magnitude).

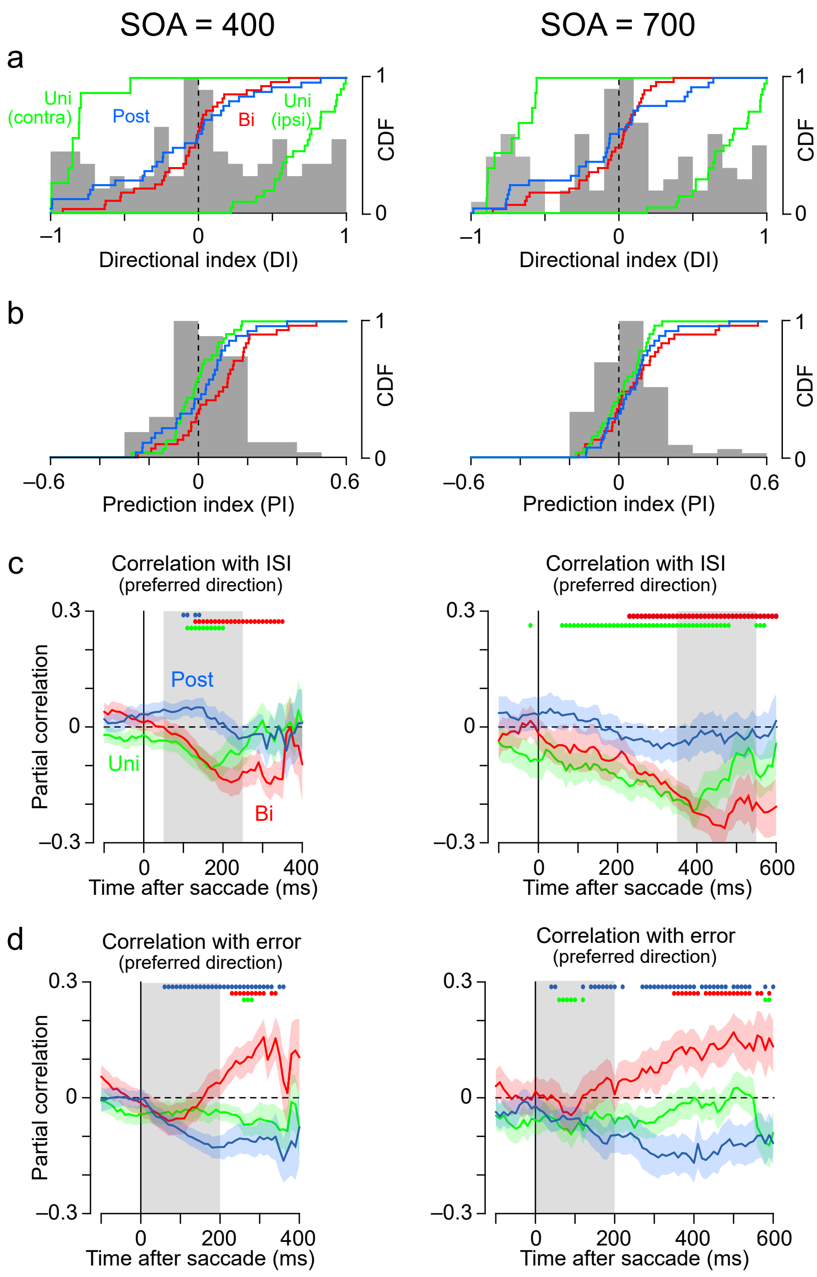

Supplementary Figure 5 | Response properties for different SOAs. (**a**) Distribution of the DI for the 400-ms (*left*) and 700-ms (*right*) SOA conditions. (**b**) Distribution of the PI for different SOA conditions. (**c**) Time courses of the partial correlation coefficients (mean ± s.e.) between neuronal activity and the time of the next saccade (intersaccadic interval, ISI) for different SOA conditions. Similar to the 550-ms SOA condition (Fig. 3e), the partial correlation coefficients significantly differed from zero for both Unilateral neurons (400-ms SOA, measured during 50–250 ms, −0.09 ± 0.19, one sample *t*-test, two-sided, $t_{32} = -2.62$, $P = 0.01$; 700-ms SOA, measured during 350–550 ms, −0.16 ± 0.28, $t_{32} = -3.40$, $P = 0.002$) and Bilateral neurons (400-ms SOA, −0.09 ± 0.17, $t_{32} = -2.84$, $P = 0.008$, 700-ms SOA, −0.25 ± 0.25, $t_{32} = -5.85$, $P = 1.7 \times 10^{-6}$). (**d**) Partial correlation coefficients (mean ± s.e.) between neuronal activity and the temporal error for different SOA conditions. The partial correlation coefficients for Postsaccade neurons significantly differed from zero in trials with a 400-ms SOA (measured during 0–200 ms, −0.08 ± 0.13, one sample *t*-test, two-sided, $t_{28} = -3.53$, $P = 0.002$) but not in trials with a 700-ms SOA (−0.05 ± 0.17, $t_{28} = -1.70$, $P = 0.10$).

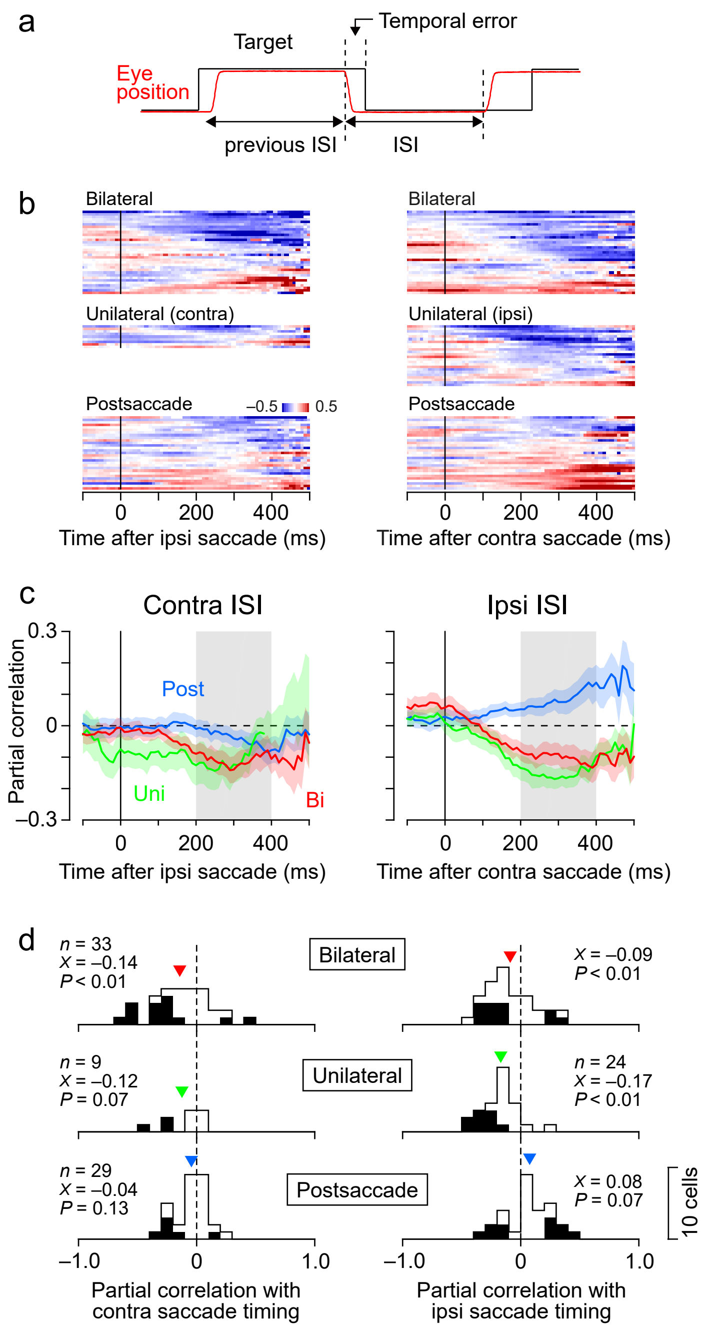

Supplementary Figure 6 | Correlation with the timing of the next saccade in different directions. (**a**) Schematic of the partial correlation analysis with the next saccade timing. The Pearson's partial correlation between the trial-by-trial neuronal activity at every 200-ms interval (10 ms step) following saccades and the time of the next saccade (intersaccadic interval, ISI) was calculated by controlling the temporal error and the preceding ISI during synchronized saccades (SOA, 550 ms). (**b**) Time courses of the partial correlation coefficients in individual neurons calculated for ipsilateral and contralateral saccades. Data of Unilateral neurons are shown for saccades in the preferred direction only, resulting in a different number of neurons in each direction. (**c**) Time courses of the mean (± s.e.) of the partial correlation between neuronal activity and the ISI during synchronization. (**d**) Distributions of the partial correlation coefficients measured during 200–400 ms following saccades (gray shade in **c**) in different directions. The black bar indicates a statistically significant value (permutation test, $P < 0.05$). In the population, a significant correlation was found for Bilateral neurons in both saccade directions (one sample t-test, two-sided, contralateral, $t_{32} = -3.27$, $P = 0.003$, ipsilateral, $t_{32} = -2.58$, $P = 0.01$) and for Unilateral neurons with ipsilateral saccade timing ($t_{23} = -5.26$, $P = 2.4 \times 10^{-5}$; contralateral, $t_8 = -2.11$, $P = 0.07$), but not for Postsaccade neurons (contralateral, $t_{28} = -1.57$, $P = 0.13$, ipsilateral, $t_{28} = 2.00$, $P = 0.07$). The coloured inverted triangle denotes the mean value.

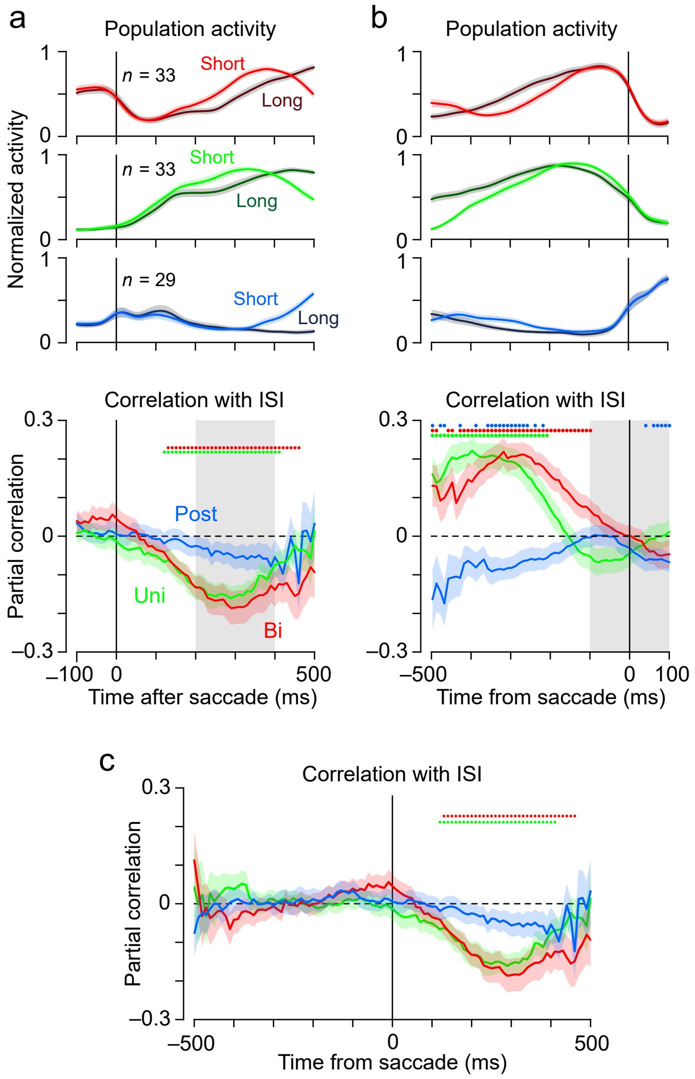

Supplementary Figure 7 | Correlation with the intersaccadic interval in different response alignment. (**a**) The time course of the population activity and the partial correlation coefficient for the next saccade timing aligned with the previous saccades (same data as in Fig. 3c–e). (**b**) Population activity and the partial correlation coefficient for the ISI calculated for the data aligned with the next saccades. Since the peak of the preparatory activity aligned with the next saccades was similar between trials with early and late saccades, the partial correlation coefficients around the time of saccade (± 100 ms, gray shading) were not different from zero (one sample ***t***-test, two-sided, $P > 0.05$). There were positive partial correlations at earlier time periods for both Unilateral and Bilateral neurons, reflecting the difference in ramping activity between trials with short versus long ISIs. Note that the sign of the partial correlation is reversed due to the difference in alignment because the faster increase in preparatory activity in trials with shorter ISI (shown in **a**) corresponds the shorter lead time of the ramping activity (in **b**). (**c**) The time course of the partial correlation shown in **a** are extended backward in time, showing that there was no significant correlation between the neuronal activity and the two later saccade timing. In all panels, data are presented as mean ± s.e.

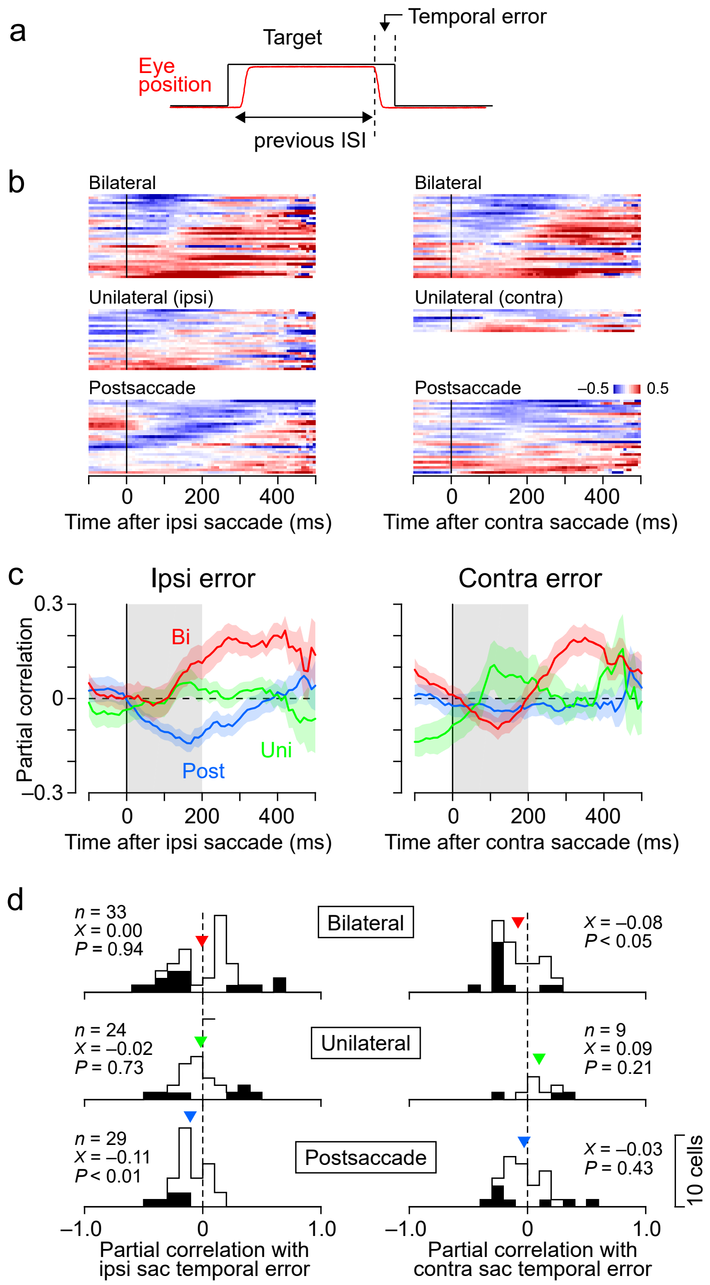

Supplementary Figure 8 | Correlation with the temporal error for different saccade directions. (**a**) Schematic of the partial correlation analysis for temporal error. The Pearson's partial correlation between the trial-by-trial neuronal activity at every 200-ms interval (10 ms step) and temporal error was calculated by controlling the preceding ISI during synchronized saccades (SOA, 550 ms). (**b**) Time courses of the partial correlation coefficients in individual neurons calculated for ipsilateral and contralateral saccades. Data of Unilateral neurons are shown for saccades in the preferred direction only. (**c**) Time courses of the means (± s.e.) of partial correlation between neuronal activity and temporal error for the three types of neurons. (**d**) Distributions of the partial correlation coefficients for temporal error. A significant correlation was found for Postsaccade neurons during ipsilateral saccades (one sample *t*-test, two-sided, $t_{28} = -4.14$, $P = 2.9 \times 10^{-4}$; contralateral, $t_{28} = -0.81$, $P = 0.43$) and for Bilateral neurons during contralateral saccades ($t_{32} = -2.48$, $P = 0.019$; ipsilateral, $t_{32} = -0.07$, $P = 0.94$), but not for Unilateral neurons (ipsilateral, $t_{23} = 0.35$, $P = 0.73$; contralateral, $t_8 = 1.35$, $P = 0.21$). The convention of the graph is the same as that in Supplementary Fig. 6d.

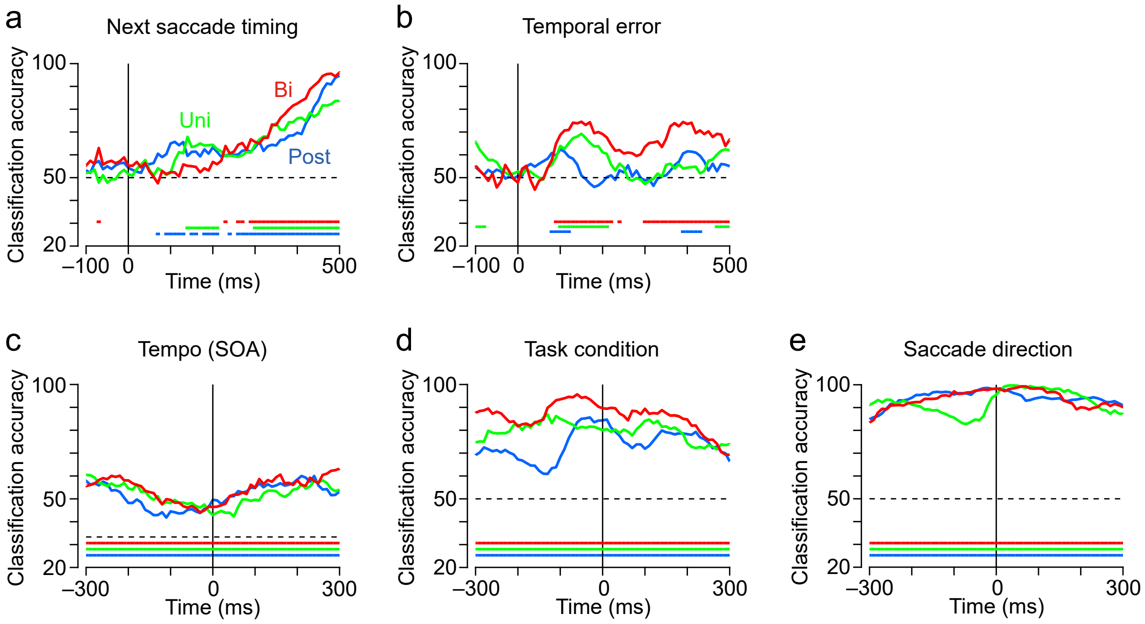

Supplementary Figure 9 | Accuracy of the decoding analysis. (**a**) Classification accuracy for the timing of the next saccade by constructing population decoder for each neuron group, using the same data set for the partial correlation analysis in Fig. 3e (synchronized saccade task, preferred direction, 550 ms SOA). The coloured bars below the traces indicate when the decoder classified trials significantly above chance (permutation test, $P < 0.05$, maximum correlation coefficient classifier). Decoder constructed from Bilateral and Unilateral neurons successfully discriminated the timing of saccades, consistent with the results of the partial correlation analysis shown in Fig. 3e. Decoder for Postsaccade neurons also showed good results, probably due to the inverse correlation of successive saccade timing during synchronization (which was controlled in the partial correlation analysis). (**b**) Performance of the decoder for temporal error using the same data set as the partial correlation analysis in Fig. 4e (synchronized saccade task, preferred direction, 550 ms SOA). (**c**) Performance of the decoder for tempo (SOA) around the time of synchronized saccades in the preferred direction. (**d**) Performance of the decoder for task condition constructed from the data for both the synchronized and reactive saccade tasks. (**e**) Performance of the decoder for saccade direction constructed from the data for synchronized saccades. Saccade direction could be decoded reliably from the population activity of all types of neurons, consistent with the ANOVA results shown in Supplementary Fig. 2b.

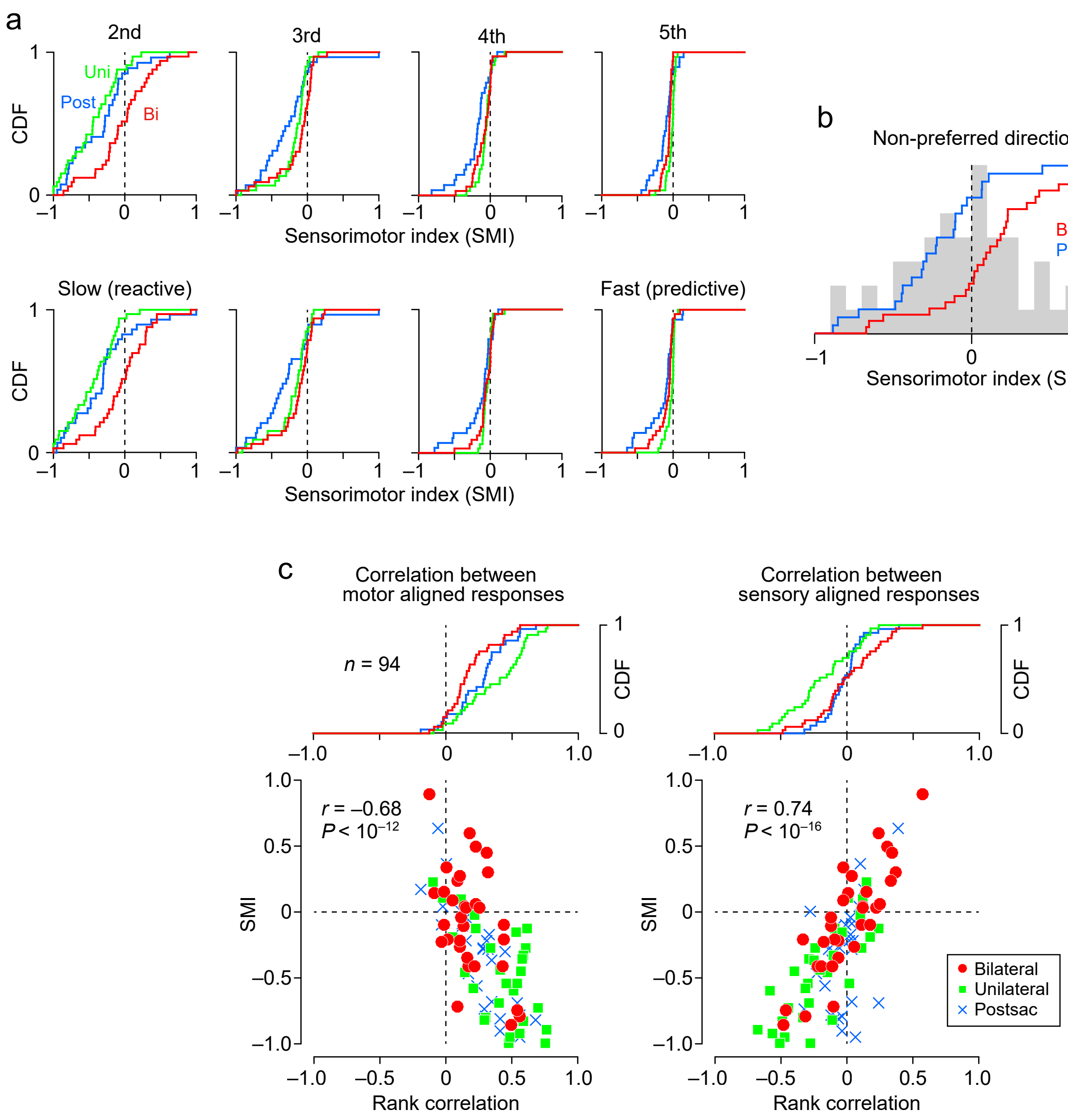

Supplementary Figure 10 | Comparison of neuronal activity in response to sensory versus motor events. (**a**) The sensorimotor indices (SMIs) were computed for the 2nd through the 5th saccades in the preferred direction (upper panels). The same data were separated into four groups according to temporal error (saccade latency), and the SMI for each group was also computed (lower panels). When the target order was early and the temporal error was large (i.e., reactive saccades), the activity of Bilateral neurons (red lines) tended to be more aligned with the target than saccade, resulting in larger SMI values. When the target order was late and the temporal error was small (synchronized saccades), the SMI value became close to zero, indicating that the magnitude of neuronal activity was comparable between different alignments of the data. (**b**) Distributions of the SMI measured for saccades in the non-preferred direction for Bilateral and Postsaccade neurons. (**c**) *Left*: Distribution of the rank correlation coefficients between the spike density profiles (± 275 ms) for early reactive (2nd saccade) and later synchronized saccades (> 5th) aligned with saccade. *Right*: Distribution of the rank correlation coefficients calculated for the same data but aligned with the target onset. For Unilateral neurons, the rank correlation coefficients were large for the data aligned with saccades but were small for the data aligned with the target onset, indicating that they exhibited activity associated with saccades irrespective of the order of saccade in the sequence. By contrast, for Bilateral neurons, the correlation for the data aligned with saccades was the smallest among the three types of neurons but that for the data aligned with the target onset was the largest. These coefficient values were closely correlated with the SMI measured for the second saccades (motor aligned, $r = -0.68$, $P = 1.5 \times 10^{-13}$; sensory aligned, $r = 0.74$, $P = 6.6 \times 10^{-17}$).

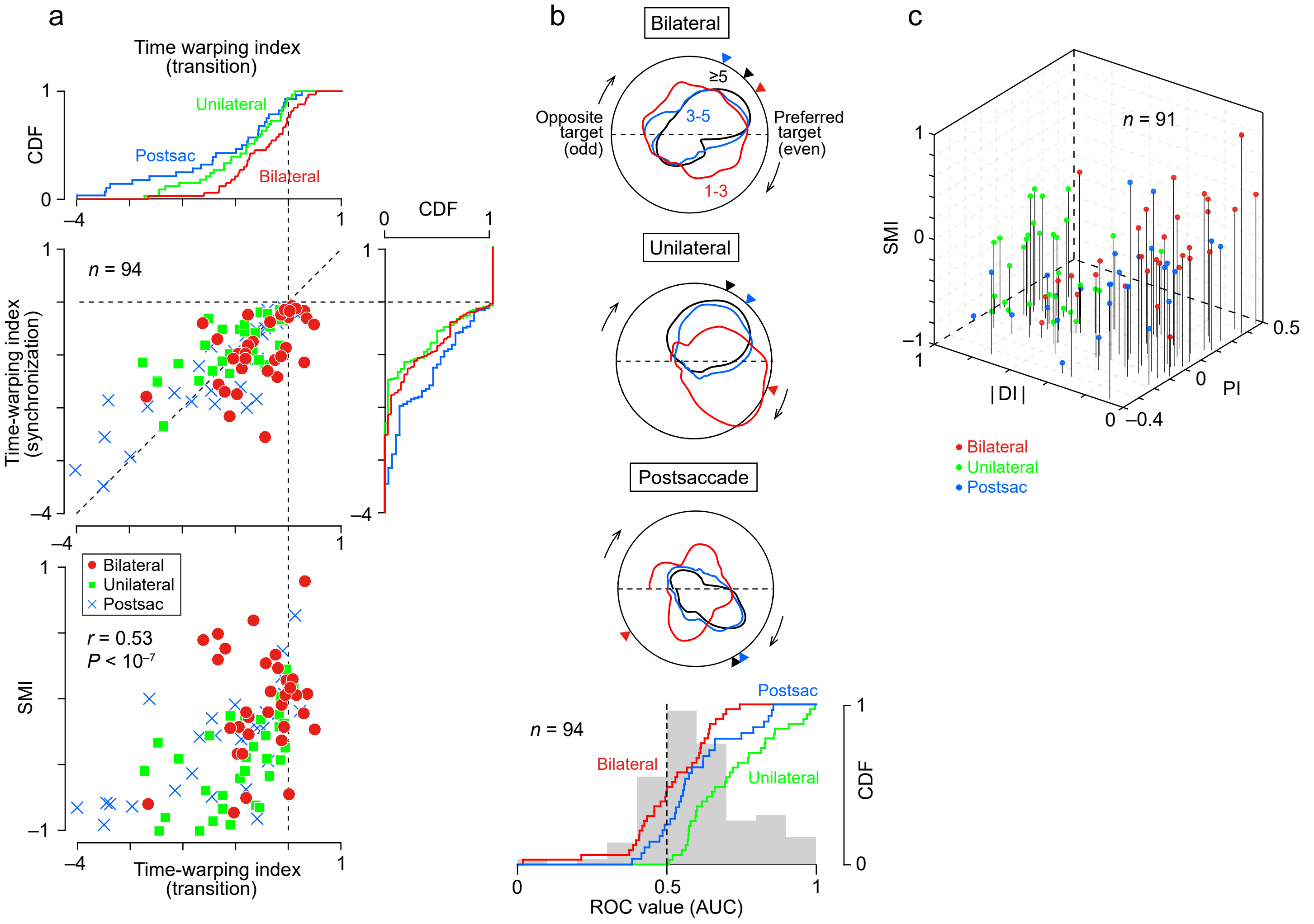

Supplementary Figure 11 | Comparison of neuronal activity during transition and synchronized saccades. (**a**) Comparison of the time-warping index (TWI) calculated for early reactive saccades (transition, a 700-ms period starting from 200 ms before the 2nd target onset, Supplementary Methods) with that calculated for synchronized saccades (synchronization, a 700-ms period starting from 350 ms before the sixth and subsequent target onset). Only Bilateral neurons exhibited different TWIs between the transition and the synchronization phases (middle panel, paired *t*-test, two-sided, $t_{32} = 3.19$, $P = 0.003$; Unilateral neurons, $t_{32} = -1.69$, $P = 0.10$; Postsaccade neurons, $t_{27} = 0.15$, $P = 0.88$). The TWI during transition closely correlated with the SMI measured for the second saccades (bottom, $r = 0.53$, $P = 4.5 \times 10^{-8}$). (**b**) Polar plot represents the time course of population activity for the initial five (i.e., two cycles) and later targets in the sequence (SOA 550 ms). For each type of neuron, the normalized population activity aligned with the target onset (eccentricity) are plotted as a function of time (angle). For all panels, the even-numbered target is the preferred direction and is shown to the right. The outer triangles indicate the orientation of the mean vector computed for each cycle. Note that the timing of activity for the first (red curve), second (blue), and the later cycles (black) were relatively consistent in Bilateral neurons, but changed significantly in Unilateral neurons. Bottom panel illustrates the distributions of the AUC value obtained from the ROC analysis that compared the time course of neuronal activity between the initial and the later cycles for individual neurons (Supplementary Methods). (**c**) Relationship of the three indices that were used to characterize the activity of individual neurons.

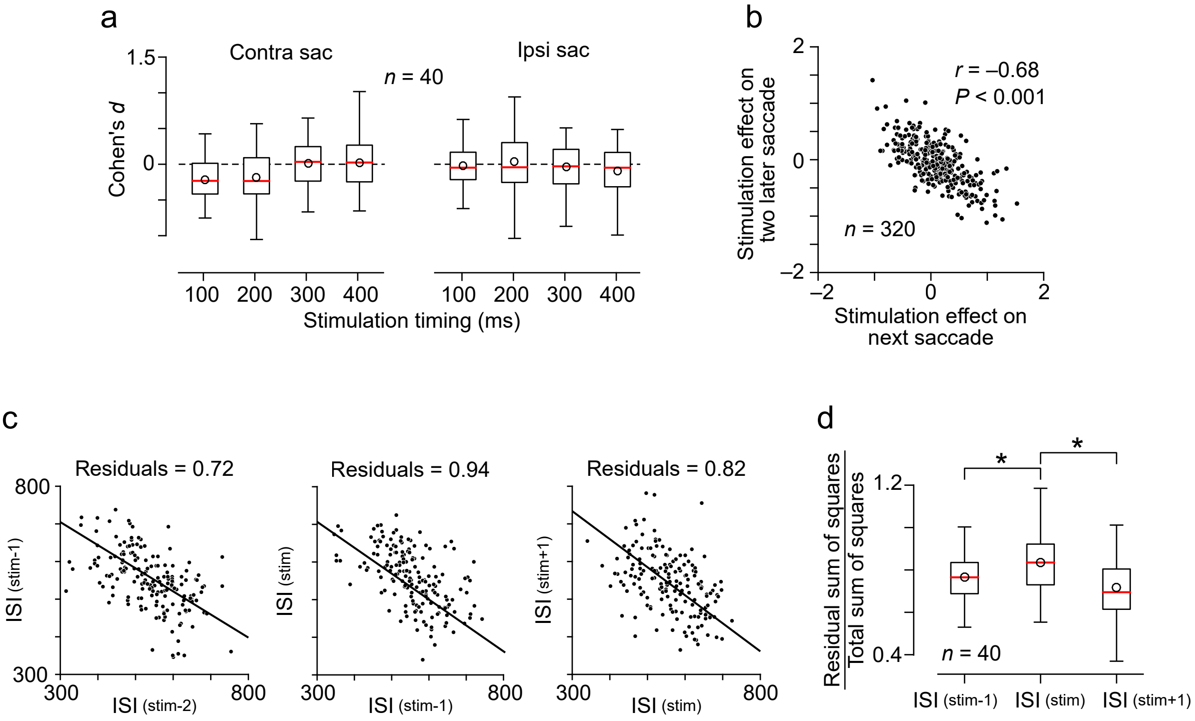

Supplementary Figure 12 | Effect of electrical stimulation on the timing of the two later saccade. (**a**) The box-whisker plot shows the median, quartiles, and range of the effect size for the timing of the two later saccade following electrical stimulation. Circle indicates the mean value. Same graph format as in Figure 6b. (**b**) Comparison of the effect size for the timing of the first versus second saccades after electrical stimulation in each condition. The strong negative correlation (r = –0.68, P = 2.0 × 10^{-45}) suggests that the stimulation effect on the two later saccade may be due to the inverse correlation of successive saccade timing during synchronization, rather than the direct effect of electrical stimulation. (**c**) Relationship of successive ISIs in a representative session. Black dots represent ISIs in trials with electrical stimulation and the oblique line represents a regression line calculated for randomly interleaved non-stimulation control. Each panel compares the successive ISIs before (left panel), during (middle), and after (right) electrical stimulation. The stimulation effect was quantified as the degree of violation from the expected inverse correlation (black line) by calculating the normalized residuals (squared sum of the residual from the control data divided by the squared sum of the deviation of the stimulation data). (**d**) Summary of the normalized residuals before (stim−1), during (stim), and after (stim+1) electrical stimulation. The box-whisker plot indicates the median, quartiles, and range of the data. Circle indicates the mean value. The residuals for ISI_{stim} were significantly larger than those for ISI_{stim-1} (Tukey test, P = 0.009, n = 40), while there was no significant difference between the residuals for ISI_{stim-1} and ISI_{stim+1} (P = 0.09). These results suggest that electrical stimulation directly changed the timing of the next saccade, but the apparent stimulation effect on the two later saccade was mostly due to the behavioural adjustment during synchronization.

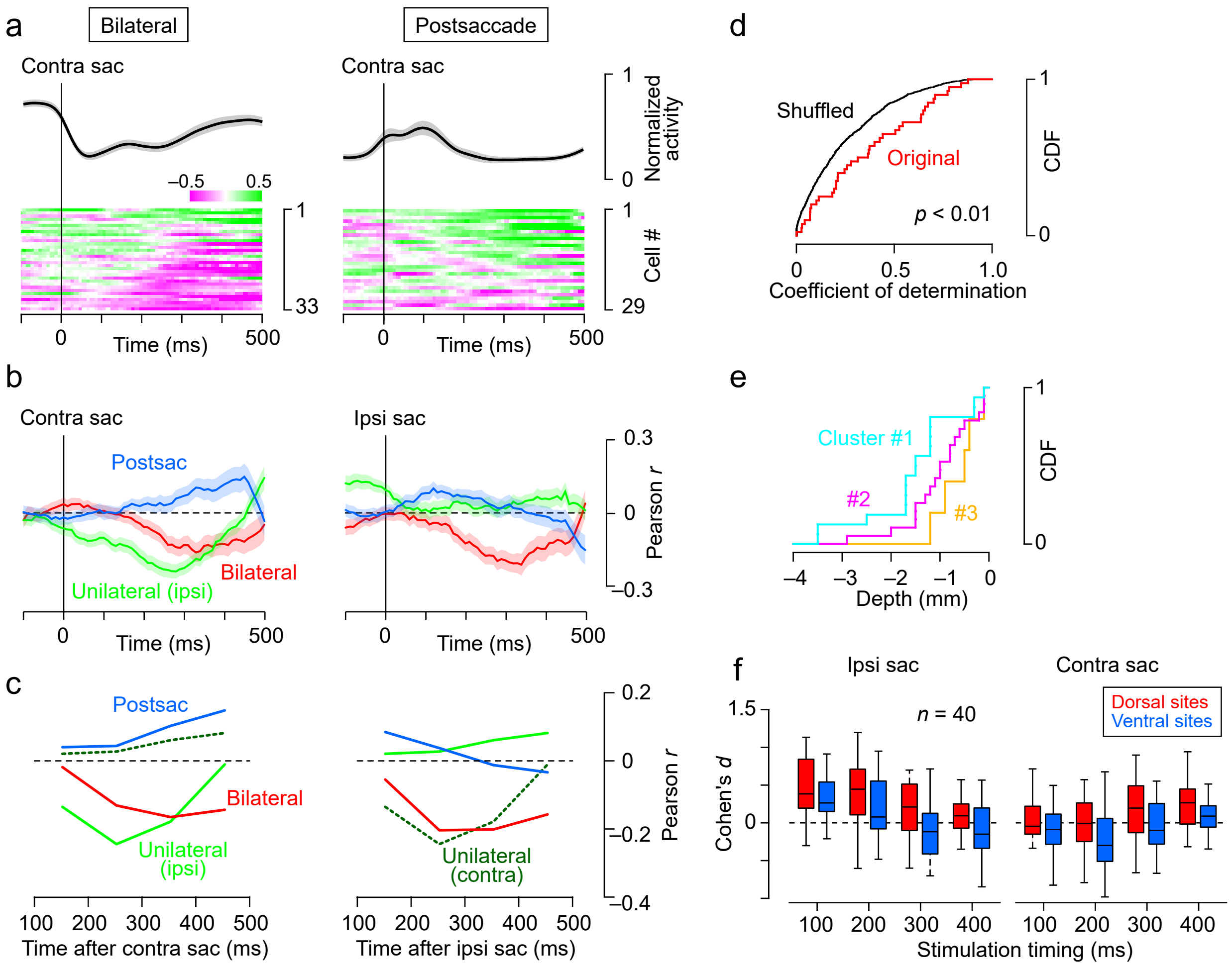

Supplementary Figure 13 | Estimate of neuronal impacts at the time of electrical stimulation on the next saccade timing. (**a**) Simple correlation coefficients between neuronal activity measured every 100 ms in individual neurons and the ISI. The data are aligned with contralateral saccade and reveal the correlation with the timing of the subsequent ipsilateral saccade. The above lines represent the normalized population activity (mean ± s.e.). (**b**) Time courses of simple correlation coefficients (mean ± s.e.) computed for different saccade directions. Negative values indicate that the greater the neuronal activity, the more the next saccade is promoted (or the shorter the ISI). Since most Unilateral neurons showed a preference for ipsilateral saccades (Fig. 2a), correlations of Unilateral neurons with different directional preferences were calculated for the preferred (non-preferred) directions and shown for ipsilateral (contralateral) saccades. (**c**) Estimated impacts of neuronal activity on subsequence saccade timing at the time of electrical stimulation. Pearson's correlation coefficients were computed at different stimulation times (100 ms duration) following synchronized saccades. For the analysis in Fig. 6e, we assumed that the effects of electrical stimulation could be accounted for by the combination of signals in different type of neurons (Methods). (**d**) Distribution of the coefficient of determination (r^2) for fitting the data of electrical microstimulation. The black curve represents the null distribution generated by the permutation method (1,000 repeats). (**e**) Cumulative distributions of stimulation sites along the dorsoventral axis for the three groups. The colours match the groupings in Fig. 6c–e. (**f**) Effect of electrical stimulation separated for dorsal and ventral stimulation sites. The box-whisker plot indicates the median, quartiles, and range of the effect size. Stimulation of dorsal sites slowed, but stimulation of ventral sites facilitated, the next saccade.

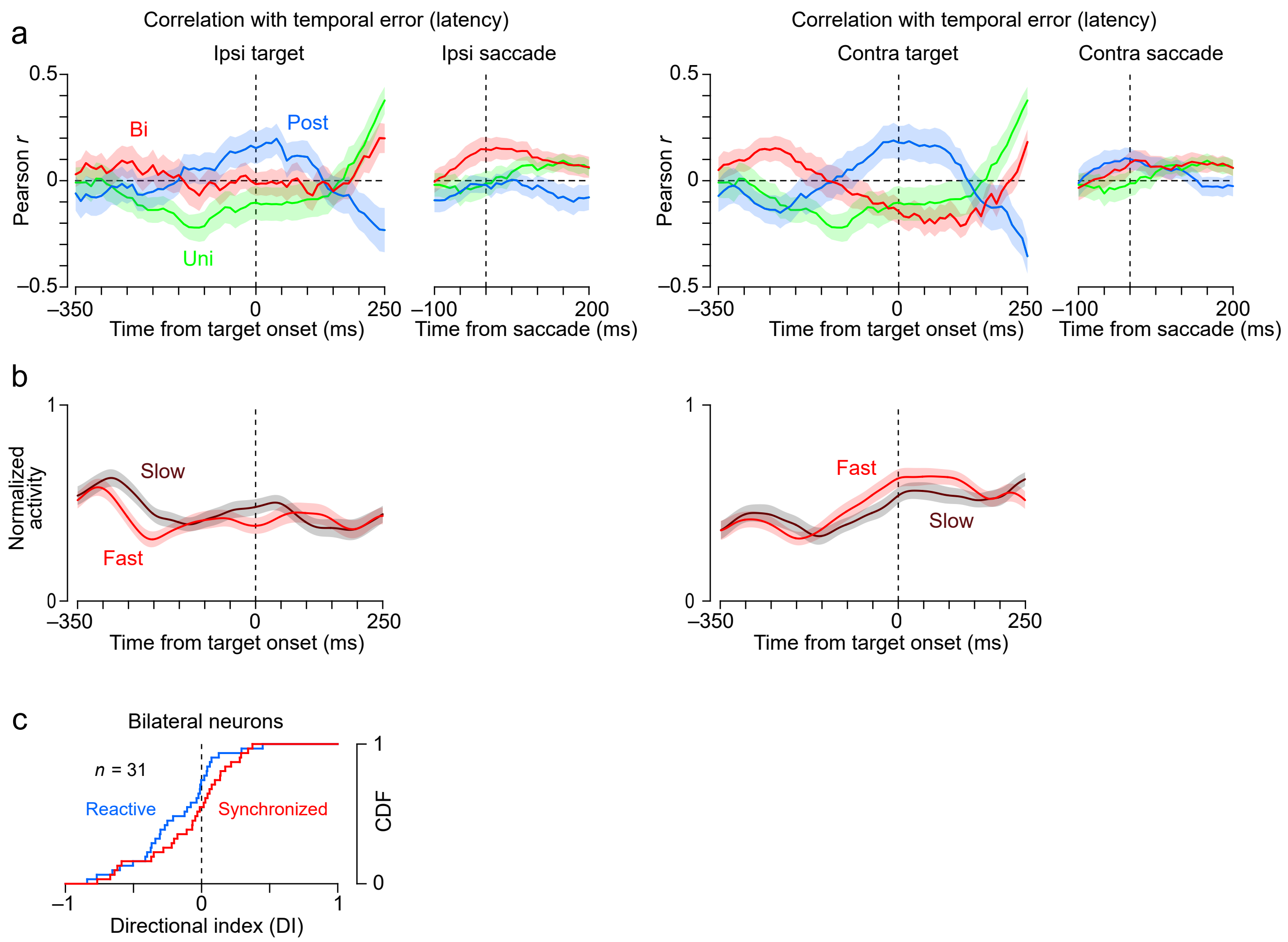

Supplementary Figure 14 | Time course of neuronal activity and simple correlation coefficients during reactive saccades. (**a**) *Left*: Time courses of simple correlation coefficients (mean ± s.e.) around the time of target onset computed for reactive saccades. Negative values indicate that the greater the neuronal activity, the more the following saccade is promoted. *Right*: Correlation coefficients calculated for the data aligned with saccades. (**b**) Normalized activity of Bilateral neurons in one-third of the trials with early and late saccades in the reactive saccade task (mean ± s.e.). Note that these neurons exhibited ramping activity for contralateral reactive saccades only, while they showed preparatory activity for synchronized saccades in both directions (Fig. 2a and Supplementary Fig. 3). (**c**) Distribution of the directional index (DI) of Bilateral neurons computed for synchronized (red) and reactive (black) saccades. The DI in the reactive saccade task was significantly smaller than zero (one sample *t*-test, two-sided, t_{30} = 3.56, P = 0.001), and was significantly different from that in the synchronized saccade task (paired *t*-test, two-sided, t_{30} = 3.51, P = 0.001).

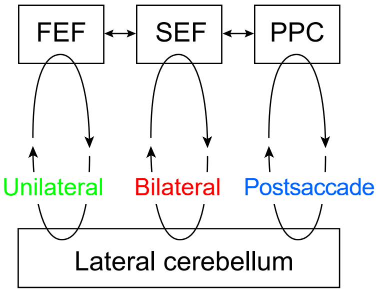

Supplementary Figure 15 | A hypothetical diagram incorporating multiple cerebrocerebellar loops for the generation of synchronized saccades. Unilateral neurons are involved in the link with the FEF or superior colliculus, which regulates the timing of the next saccade. Bilateral neurons participate in the network with the SEF, which represents the internal rhythm of periodic targets. Postsaccade neurons contribute to the link with the PPC and monitor the temporal error of the current saccade to update the internal rhythm. FEF, the frontal eye fields; PPC, the posterior parietal cortex; SEF, the supplementary eye field.

References

1. Werner G, Mountcastle VB. The variability of central neural activity in a sensory system, and its implications for the central reflection of sensory events. *J Neurophysiol* **26**, 958-977 (1963).

2. Schreiner RC, Essick GK, Whitsel BL. Variability in somatosensory cortical neuron discharge: effects on capacity to signal different stimulus conditions using a mean rate code. *J Neurophysiol* **41**, 338-349 (1978).

3. Merchant H, Zarco W, Perez O, Prado L, Bartolo R. Measuring time with different neural chronometers during a synchronization-continuation task. *Proc Natl Acad Sci U S A* **108**, 19784-19789 (2011).

4. Meyers EM. The neural decoding toolbox. *Front Neuroinform* **7**, 8 (2013).

5. Perez O, Kass RE, Merchant H. Trial time warping to discriminate stimulus-related from movement-related neural activity. *J Neurosci Methods* **212**, 203-210 (2013).
