# Supplementary material for: Neural signals regulating motor synchronization in the primate deep cerebellar nuclei
Source: Nat Commun. 2022 May 6;13:2504. doi: 10.1038/s41467-022-30246-2 (PMC9076601; doi:10.1038/s41467-022-30246-2)
Supplement: Supplementary file 3 — Description of Additional Supplementary Files [file 41467_2022_30246_MOESM3_ESM.pdf]

Supplementary Movie 1

Description: Behavioural performance during recording of a Unilateral neuron.

Supplementary Movie 2

Description: Behavioural performance during recording of a Bilateral neuron.
