# Supplementary material for: Neural signals regulating motor synchronization in the primate deep cerebellar nuclei
Source: Nat Commun. 2022 May 6;13:2504. doi: 10.1038/s41467-022-30246-2 (PMC9076601; doi:10.1038/s41467-022-30246-2)
Supplement: Supplementary file 6 — Reporting Summary [file 41467_2022_30246_MOESM6_ESM.pdf]

nature portfolio

Corresponding author(s): Masaki Tanaka

Last updated by author(s): Apr 1, 2022

Reporting Summary

Nature Portfolio wishes to improve the reproducibility of the work that we publish. This form provides structure for consistency and transparency in reporting. For further information on Nature Portfolio policies, see our Editorial Policies and the Editorial Policy Checklist.

Statistics

For all statistical analyses, confirm that the following items are present in the figure legend, table legend, main text, or Methods section.

n/a	Confirmed	
☐	☒	The exact sample size (n) for each experimental group/condition, given as a discrete number and unit of measurement
☐	☒	A statement on whether measurements were taken from distinct samples or whether the same sample was measured repeatedly
☐	☒	The statistical test(s) used AND whether they are one- or two-sided<br>*Only common tests should be described solely by name; describe more complex techniques in the Methods section.*
☐	☒	A description of all covariates tested
☒	☐	A description of any assumptions or corrections, such as tests of normality and adjustment for multiple comparisons
☐	☒	A full description of the statistical parameters including central tendency (e.g. means) or other basic estimates (e.g. regression coefficient) AND variation (e.g. standard deviation) or associated estimates of uncertainty (e.g. confidence intervals)
☐	☒	For null hypothesis testing, the test statistic (e.g. F, t, r) with confidence intervals, effect sizes, degrees of freedom and P value noted<br>*Give P values as exact values whenever suitable.*
☒	☐	For Bayesian analysis, information on the choice of priors and Markov chain Monte Carlo settings
☒	☐	For hierarchical and complex designs, identification of the appropriate level for tests and full reporting of outcomes
☐	☒	Estimates of effect sizes (e.g. Cohen's d, Pearson's r), indicating how they were calculated

Our web collection on statistics for biologists contains articles on many of the points above.

Software and code

Policy information about availability of computer code

Data collection	TEMPO system (Reflective Computing, release 2020 or older)<br>ASD (Alpha Omega Engineering, version 3.00)
Data analysis	MATLAB (Mathworks, version 2016)

For manuscripts utilizing custom algorithms or software that are central to the research but not yet described in published literature, software must be made available to editors and reviewers. We strongly encourage code deposition in a community repository (e.g. GitHub). See the Nature Portfolio guidelines for submitting code & software for further information.

Data

Policy information about availability of data

All manuscripts must include a data availability statement. This statement should provide the following information, where applicable:

- Accession codes, unique identifiers, or web links for publicly available datasets
- A description of any restrictions on data availability
- For clinical datasets or third party data, please ensure that the statement adheres to our policy

All data analysed here are included in this article and its supplementary information files. Numerical data for each figure and supplemental figures are provided in the Source Data file.

Field-specific reporting

Please select the one below that is the best fit for your research. If you are not sure, read the appropriate sections before making your selection.

☒ Life sciences ☐ Behavioural & social sciences ☐ Ecological, evolutionary & environmental sciences

For a reference copy of the document with all sections, see nature.com/documents/nr-reporting-summary-flat.pdf

Life sciences study design

All studies must disclose on these points even when the disclosure is negative.

Sample size	Quantitative analysis was performed on 95 cerebellar nuclear neurons in two monkeys for which we were able to examine neuronal activity with a sufficient number (>200) of predictive saccades. Effects of electrical stimulation were assessed on the data from 40 stimulation sites. No statistical method was used to determine the sample size. Sample size for each experiment was determined based on previous reports of electrophysiology experiments with single electrodes in behaving animals.
Data exclusions	No data exclusions. Details of data collection are described in Methods and Results.
Replication	All findings were statistically evaluated. Behavioral and physiological observations were robust and clearly present in both animals as stated in the Results. Once we identified the relevant sites in the deep cerebellar nuclei, we were able to encounter task-related neurons in almost every recording session, but it was often difficult to isolate single neurons for long period of time in behaving monkeys.
Randomization	This study does not contain clinical data. Correlation analysis with behavior was performed at the neuron level, not across animals, and sampling of task-related neurons cannot be artificially controlled.
Blinding	This study does not contain clinical data. Blinding is not relevant for this study because all data were obtained from experimental animals and data were statistically evaluated without any subjective measures.

Reporting for specific materials, systems and methods

We require information from authors about some types of materials, experimental systems and methods used in many studies. Here, indicate whether each material, system or method listed is relevant to your study. If you are not sure if a list item applies to your research, read the appropriate section before selecting a response.

Materials & experimental systems

n/a	Involved in the study	
☒	☐	Antibodies
☒	☐	Eukaryotic cell lines
☒	☐	Palaeontology and archaeology
☐	☒	Animals and other organisms
☒	☐	Human research participants
☒	☐	Clinical data
☒	☐	Dual use research of concern

Methods

n/a	Involved in the study	
☒	☐	ChIP-seq
☒	☐	Flow cytometry
☐	☒	MRI-based neuroimaging

Animals and other organisms

Policy information about studies involving animals; ARRIVE guidelines recommended for reporting animal research

Laboratory animals	Two male Japanese monkeys (Macaque fuscata), 7–9 years old, 8–9 kg
Wild animals	No wild animals were used in this study. All animals were raised for experiments and were provided by the National Bio-resource Project in Japan.
Field-collected samples	No field collected samples were used in the study.
Ethics oversight	All experimental protocols were evaluated and approved in advance by the Hokkaido University Animal Care and Use Committee and were in accordance with the Guidelines for Proper Conduct of Animal Experiments (Science Council of Japan, 2006).

Note that full information on the approval of the study protocol must also be provided in the manuscript.

Magnetic resonance imaging

Experimental design

Design type	T2-weighted structural MRI for locating the recording sites.
Design specifications	Taken from anesthetized animals.
Behavioral performance measures	This is not relevant because we did not collect any behavioral data during MR scanning.

Acquisition

Imaging type(s)	Structural
Field strength	3.0T (MAGNETOM Prisma, Siemens)
Sequence & imaging parameters	3D T2-weighted images with 0.5-mm slices
Area of acquisition	Whole brain

Diffusion MRI ☐ Used ☒ Not used

Preprocessing

Preprocessing software	N/A. We did not preprocess the MRI data.
Normalization	N/A. We did not normalize MR images.
Normalization template	N/A
Noise and artifact removal	N/A. We did not remove artifact from MR images.
Volume censoring	N/A

Statistical modeling & inference

Model type and settings	N/A. We did not evaluate MR images statistically.
Effect(s) tested	N/A

Specify type of analysis: ☐ Whole brain ☐ ROI-based ☒ Both

Anatomical location(s)	ROI was located on the coronal sections underneath the recording chamber. Recording sites relative to the interaural line and the anterior commissure were also verified.
Statistic type for inference (See Eklund et al. 2016)	N/A
Correction	N/A

Models & analysis

n/a	Involved in the study
☒	☐ Functional and/or effective connectivity
☒	☐ Graph analysis
☒	☐ Multivariate modeling or predictive analysis
